# Identifying cancer-associated fibroblasts as emerging targets for hepatocellular carcinoma

**DOI:** 10.1186/s13578-020-00488-y

**Published:** 2020-10-31

**Authors:** Jie Zhang, Chaoyu Gu, Qianqian Song, Mengqi Zhu, Yuqing Xu, Mingbing Xiao, Wenjie Zheng

**Affiliations:** 1 Research Center of Clinical Medicine, Affiliated Hospital of Nantong University, 20 Xisi Road, Nantong, 226001 Jiangsu China; 2 School of Medicine, Nantong University, 19 Qixiu Road, Nantong, 226001 Jiangsu China; 3 Department of Radiology, Wake Forest School of Medicine, One Medical Center Boulevard, Winston-Salem, NC 27157 USA

**Keywords:** Cancer-associated fibroblasts, Hepatocellular carcinoma, Tumor microenvironment, Molecular target

## Abstract

The tumor microenvironment (TME) is a complex multicellular functional compartment that includes fibroblasts, myofibroblasts, endothelial cells, immune cells, and extracellular matrix (ECM) elements. The microenvironment provides an optimum condition for the initiation, growth, and dissemination of hepatocellular carcinoma (HCC). As one of the critical and abundant components in tumor microenvironment, cancer-associated fibroblasts (CAFs) have been implicated in the progression of HCC. Through secreting various growth factors and cytokines, CAFs contribute to the ECM remodeling, stem features, angiogenesis, immunosuppression, and vasculogenic mimicry (VM), which reinforce the initiation and development of HCC. In order to restrain the CAFs-initiated HCC progression, current strategies include targeting specific markers, engineering CAFs with tumor-suppressive phenotype, depleting CAFs’ precursors, and repressing the secretions or downstream signaling. In this review, we update the emerging understanding of CAFs in HCC, with particular emphasis on cellular origin, phenotypes, biological functions and targeted strategies. It provides insights into the targeting CAFs for HCC treatment.

## Background

Hepatocellular carcinoma (HCC) is the fourth leading cause of cancer-related mortality worldwide [1]. According to the latest statistics, almost 85% of HCC cases occur in developing countries such as Eastern Asia and sub-Saharan Africa, where chronic hepatitis B virus (HBV) is the most common etiology [2, 3]. In contrast, for developed countries, the primary causes of HCC are hepatitis C virus (HCV), alcoholic cirrhosis, and non-alcoholic steatohepatitis [4]. Over the past two decades, the incidence of HCC in the USA has increased twofold to threefold, mainly ascribed to the growing HCV-related cirrhosis and the prevalence of non-alcoholic fatty liver disease (NAFLD) [5, 6]. With the increasing heavy alcohol consumption, obesity, and insulin resistance (IR), more alcohol-related liver disease (ALD)-related HCC and metabolic-related HCC cases have been reported [1, 7].

During the hepatocarcinogenesis, inflammation and fibrosis are well acknowledged as the key drivers. Persistent liver damage can lead to liver fibrosis with the formation of regenerative and dysplastic nodules. Continuous cycles of such destructive–regenerative process eventually gives rise to liver cirrhosis and even carcinogenesis. Indeed, about 1/3 patients with liver cirrhosis will ultimately develop to HCC [8]. However, early diagnosis of HCC is challenging due to the hidden symptoms. Approximately 80% of HCC patients are diagnosed at advanced stage, of which the median survival is around 6–8 months. For these patients at advanced stages, treatment strategies include, liver transplantation, topical therapy, and systemic chemotherapy. Additionally, antiangiogenics and immunotherapies are also considered as effective treatment choices. Sorafenib, a small multi-tyrosine kinase inhibitor (TKI), has been approved as the first-line treatment of advanced HCC for over 10 years [9]. Another first line agent, Lenvatinib, has also been evaluated as a novel agent against advanced HCC. For patients that fail in first-line therapy, other antiangiogenic agents (e.g. regorafenib, cabozantinib, and ramucirumab) and immunotherapies (e.g. nivolumab and pembrolizumab) are recommended as second-line strategies [10–14].

Despite the current available treatment strategies, the overall prognosis of HCC remains unsatisfactory. Therefore, the underlying molecular mechanisms that drive HCC progression and metastasis still need to be investigated. Increasing studies suggest that targeting tumor cells exclusively is insufficient to improve patients’ survival, as the malignant behaviors of tumor cells can be greatly modulated by the reconstruction of tumor microenvironment (TME). Given that HCC is often initiated by fibrosis or cirrhosis, the premalignant microenvironment (PME) is considered before tumor formation, which is featured by chronic liver injury, inflammation, and fibrosis in HCC [15]. Once hepatoma occurs, TME, as the suitable environment for tumor cells, takes the place of PME to promote tumor progression [16]. TME in HCC is distinguished by profound ECM remodeling and non-tumoral stromal cells, particularly the tumor-associated stromal and immune cells, including cancer-associated fibroblasts (CAFs), B and T cells, neutrophils, endothelial cells, and tumor-associated macrophages (TAMs) [17]. The reciprocal crosstalk among these compartments of TME and HCC cells significantly reinforces proliferation, migration, metastasis and chemoresistance, as well as generation of vasculogenic mimicry (VM) and immunosuppressive induction of neoplastic cells [18, 19]. As a result, the TME has been identified as a perspective target for developing potential therapeutic agents.

## Characterization of CAFs

CAFs is a heterogeneous group of dynamical fibroblasts that infiltrates in tumor. Distinct from normal fibroblasts, CAFs exhibit a spindle-shaped morphology with large indented nucleus, Golgi complexes, and endoplasmic reticulum (ER). Thus, these CAFs could acquire enhanced metabolic activities and intensive secretory performance [20]. Structurally and functionally, CAFs fertilize the microenvironment for tumor progression by secreting various factors, including ECM proteins (e.g. collagen type-I and ectodysplasin-A), epidermal growth factor (EGF)/fibroblast growth factor (FGF) family members, pro-angiogenesis factors (e.g. hypoxic inducible factor (HIF) and platelet derive growth factor (PDGF)), chemokines (e.g. CXCL and CXCR family members), cytokines (e.g. transforming growth factor-β (TGF-β)), and different enzymes [e.g. metalloproteinases (MMPs)] [21].

The heterogeneity of CAFs is contributed by the multiple cellular precursors, such as tissue-resident quiescent fibroblasts, bone marrow-derived cells, adipocytes, pericytes and endothelial cells derived from local stromal cells with endothelial-to-mesenchymal transition (EndMT), and cancer cells undergoing epithelial–mesenchymal transition (EMT) [22]. Multiple protein markers have been reported to identify CAFs, including α-smooth muscle actin (α-SMA), fibroblast activation protein (FAP), fibroblast specific protein 1 (FSP1 or S100A4), Vimentin, and PDGF receptor (PDGFR)-α and β [23]. By contrast, specific markers for distinguishing the heterogeneous CAFs are still lacking. The most acknowledged marker α-SMA can be used to identify myofibroblast-like CAFs, vascular muscular cells, and pericytes [24, 25]. Additionally, FAP seems more specific for fibroblasts, though it is also overexpressed in a subset of CD45+ immune cells [26].

The heterogenous CAF subsets have been characterized in different tumors with distinct functions. Recent study demonstrated the existence of four CAF subsets (S1–S4) in breast cancer by concomitant analysis of six markers (CD29, FSP1, FAP, α-SMA, PDGFR-β, and Caveolin1) [27]. Further investigations showed that CAF subset S1 was associated with immunosuppressive TME by inducing regulatory T cells (T-regs) differentiation, whereas CAF subset S4 improved CD8+ T cells infiltration. In addition, four subtypes with specific phenotypic features of CAFs (A–D) were identified in pancreatic ductal adenocarcinoma (PDAC), of which subtype A could furthest enhance proliferation and chemoresistance of cancer cells [28]. Interestingly, in another study of PDAC, CAFs were stratified into two subtypes, i.e. α-SMA(+) CAFs were distributed around neoplastic cells, while α-SMA(−) CAFs localized distantly from cancer cells with stronger paracrine capability of pro-inflammatory cytokines [24].

Recently, the subtypes and biological functions of CAF are analyzed precisely by using more advanced technologies, such as single-cell sequencing and flow cytometry [29]. Some researchers isolated CAFs cells from fresh HCC tumor tissues. These CAFs are featured by the fibroblastic morphology and activated myofibroblast phenotype with FSP-1 and FAP expression [30]. Moreover, techniques that provide spatial resolution, e.g. multiplexed nucleic acid in situ hybridization and highly multiplexed antibody-based staining, are also utilized to determine whether specific CAF subtype is affected by its spatial location within tumor [31]. Recent studies have shown that the precise location may impact the CAFs subtypes with phenotypic discrepancy. For instance, CAF subtype A characterized by periostin was mainly observed at the invasive edge of primary lung tumor, crucial for the tumor capsule formation and metastasis niche. The CAF subtype B marked by myosin-11, commonly identified at the invasive front of tumor, was related to lymph node metastasis and unfavorable prognosis. Besides, podoplanin-marked CAF subtype C was distributed in the central area of tumor and involved in immunogenic responses [32]. Therefore, identifying subpopulation of CAFs on the ground of the specific role in tumor progression may contribute to innovative targeted therapies and personalized therapies against various cancer types.

## CAFs in HCC

### Heterogeneous origins of CAFs in HCC

As a remarkable feature of CAFs, heterogeneity is embodied with different origins, locations, phenotypes, and functions of cells. Hepatic CAFs commonly marked with α-SMA and FAP are widely distributed in tumor septum, fibrous capsule, and hepatic blood sinusoids. According to recent studies, CAFs in HCC can be originated from multiple cell types, including hepatic stellate cells (HSCs), HCC cells undergoing EMT, mesenchymal stromal cells (MSCs), hepatic sinusoidal endothelial cells (HSECs) undergoing EndMT, and peritumoral tissue fibroblasts (PTFs).

One typical source of hepatic CAFs is HSC, which is one major accelerator in liver fibrosis [33]. Based on the genetic cell fate mapping assays, HSCs are suggested as the dominant precursor of α-SMA(+) myofibroblasts in most types of liver diseases [33–35]. Under normal conditions, quiescent HSCs are distributed in the disse space functioning as pericytes to store Vitamin A. However, sustained liver injury leads to the activation of HSCs into myofibroblasts, along with enhanced secretion of ECM, chemokines and cytokines [36]. Through migrating to the tissue repairing site, HSCs modulates hepatic fibrogenesis, sequentially causing liver fibrosis and cirrhosis. Once hepatocarcinogenesis, activated HSCs may act as CAFs and dedicate to the formation of HCC fibrous septa and fibrous capsules.

The transformation of HSCs into CAFs is a complicated process, during which intracellular factors from stromal cells and paracrine stimuli from tumor cells are inductive factors [37]. Tan et al*.* previously reported that human HSC cell line LX2 could be converted into α-SMA(+)/FSP1(+) CAFs with exposure to tumor-derived TGF-β in vitro [38]. A recent transgenic model also suggested that the hepatocyte-derived platelet derive growth factor-C (PDGF-C) transformed HSCs into myofibroblast-like cells, which in turn produced cytokines to promote the development of HCC [39]. In addition, Zhou et al*.* indicated that HCC-derived exosomal miRNA-21 could induce the conversion of HSCs into CAFs via downregulating PTEN and activating PDK1/AKT signaling pathway, subsequently accelerating tumor growth and angiogenesis by secreting massive proinflammatory cytokines [40].

Another source of hepatic CAFs is supposed to be tumor cells. HCC cells locating near the blood sinusoid commonly present with upregulated α-SMA or FAP expression. Existing evidence implicate that CAFs with high aggressiveness are originated from HCC cells undergoing EMT. Consistently, one typical CAF marker, FAP, is also associated with the EMT phenotypes of HCC cells. Zou et al*.* indicated that hypoxic condition transformed HCC cells into CAFs-like cells with enhanced FAP expression, in parallel with hypoxia inducible factor 1α (HIF-1α) and classical EMT markers (e.g. E-cadherin, Snail, and TWIST) [41]. Moreover, HCC cells exogenously acquired CAF features with the administration of EMT-related cytokine TGF-β, characterized by remarkably increased α-SMA expression [42].

In addition to HSCs and HCC cells, there are other cell types (MSCs, HSECs, and PTFs) that could be transformed into CAFs. Bhattacharya et al*.* found that MSCs acquired the CAF-like phenotype, characterized by the increased expression of tenascin-C and CXCL12, after co-culture with HCC cell line (SK-Hep1) [43]. As some CAFs distributed in hepatic blood sinusoids, endothelial cells undergoing EndMT provide another source of CAFs in the HCC microenvironment [44]. Apart from that, PTFs can also be converted into CAFs after exposure to lysophostatidic acid (LPA) secreted by HCC cells [45]. In summary, current studies indicated that CAFs in HCC are derived from various cell types, of which HSCs are considered the principal origin. However, other potential sources of CAFs like bone marrow-derived cells and portal fibroblasts (PFs) remain controversial in HCC.

### The activation of CAFs in HCC

As numerous evidences support the indispensable roles of CAFs in HCC growth and metastasis, the cancer cells also fertilized the proliferation and activation of CAFs as feedback. Once activated by corresponding stimuli from cancer cells or TME, CAF progenitors acquire CAF phenotypes and secrete plentiful factors to reinforce their tumor-supporting activities, thereby completing the positive feedback of CAF-HCC cells loop. Such bi-directional activation between cancer cells and stromal cells is critical to cancer progression.

Mazzocca et al. demonstrated that LPA secreted from HCC cells reinforced trans-differentiation of PTFs into a CAF-like myofibroblastic phenotype, which in turn facilitated proliferation, migration and invasion of HCC cells. As mentioned above, PDGF-C provoked HSCs into precursors of CAFs with abundant cytokine secretions, ultimately accelerating the progression of HCC [39]. TGF-β secreted by CAFs and HSCs has been unequivocally implicated in the malignant phenotypes of HCC cells. Giannelli et al. illustrated that TGF-β signaling advanced HCC progression by both intrinsic and extrinsic pathways from activated CAFs in TME [46]. Consistently, Mazzocca et al*.* further elucidated that inhibition of TGF-β could attenuate CAF proliferation by downregulating connective tissue growth factor (CTGF) levels, leading to a significant reduction of HCC growth and dissemination [47]. Recently, STMN1 was found to mediate the intricate crosstalk between HCC cells and CAFs. When co-cultured with HCC cells, HSCs were endowed with CAFs properties and secrete hepatocyte growth factor (HGF), thereby enhancing STMN1 expression of HCC cells through the MET pathway. In turn, upregulation of STMN1 in HCC cells activated HSCs to acquire the CAFs phenotypes [48]. Besides, HCC cells-secreted tissue inhibitor of metalloproteinase 1 (TIMP-1), an endogenous inhibitor for MMPs, has been reported to accelerate cancer progression by initiating the transformation of liver fibroblasts into CAFs. TIMP1 initiated immortalized fibroblasts into CAF-like phenotypes, characterized by elevated expression of CAF markers and enhanced proliferative activities [49]. Currently, it has been recognized that specific sub-populations of CAFs could facilitate cancer stemness by inducing proliferation of cancer stem cells (CSCs) and de-differentiation of cancer cells in a paracrine-dependent pathway. As feedback, CSCs could in turn release specific factors to maintain the activated state of CAFs [50]. Furthermore, Mano et al*.* identified bone morphogenetic Protein-4 (BMP4) as one key factor involving in the activation of CAFs [51]. Intriguingly, exogenous BMP4 exerted no direct effects on HCC invasion, instead triggering more cytokine production of CAFs in a paracrine manner.

### Biological functions of CAFs in HCC progression

**Fig. 1:**
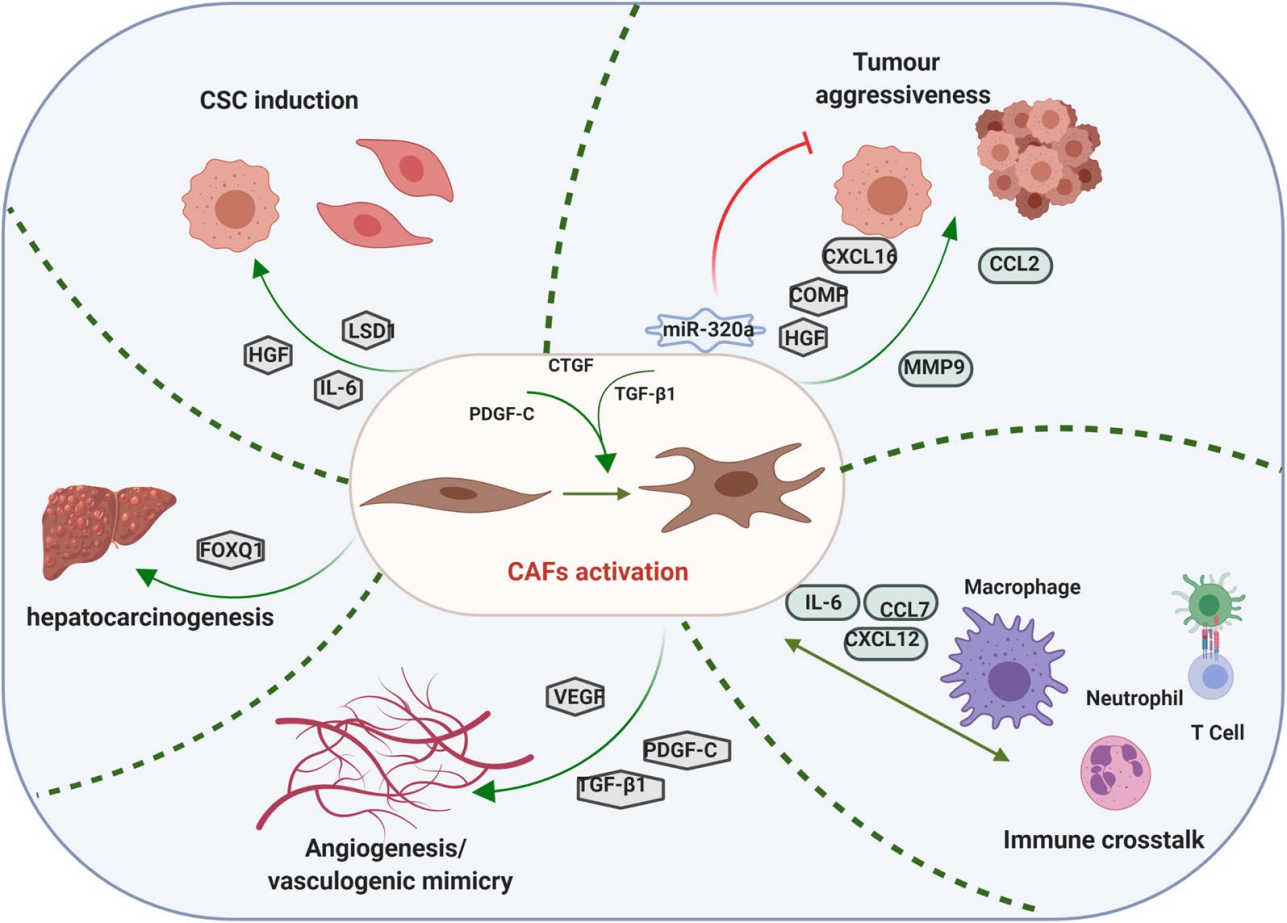
Effects of cancer-associated fibroblasts on HCC. CAFs could contribute to HCC initiation and progression by various ways, including ECM remodeling, enhancing HCC cells stemness, accelerating angiogenesis and vasculogenic mimicry (VM), as well as inducing immunosuppression. COMP, cartilage oligomeric matrix protein; ECM, extracellular matrix; VM, vasculogenic mimicry; HGF, hypoxic growth factor; PDGF, platelet derive growth factor; EMT, epithelial–mesenchymal transition; PDGFR, platelet-derived growth factor receptor; CTGF, connective tissue growth factor; CSCs, cancer stem cells; VEGF, vascular endothelial growth factor

It is noteworthy that CAFs play critical roles in the HCC progression by both direct and indirect interactions with HCC cells. CAFs have been shown to guide the collective tumor cell migration and invasion. Recent study found that the CAFs migrated through the ECM while dragging tumor cells, which was mediated by heterophilic adhesion involving N-cadherin at the CAF membrane and E-cadherin at the cancer cells [52]. Moreover, CAFs secrete multiple types of ECM proteins, growth factors, cytokines and extracellular vesicles (Table 1). With these secretions, CAFs are implicated in modulating ECM, facilitating new vessels, suppressing the anti-tumor immunity, and enhancing EMT and stem feature of HCC cells, which ultimately benefit for HCC initiation with malignant phenotypes (Fig. 1).

**Table 1:** Effects of CAFs-secreted factors on HCC

CAFs secretion	Molecules	Effect on HCC	Mechanism	References
ECM proteins	BMP4	Invasion	Trigger cytokines through SMAD pathway	[51]
	COMP	Proliferation, migration, invasion,	Promote EMT process	[30]
	MMP9	Invasion, metastasis	–	[53]
Growth factors	HGF	Proliferation	–	[54]
		Stemness	Facilitate c-Met/ FRA1/ HEY1 signaling and STAT3 signaling	[55, 56]
	TGF-β	Induce VM	VE-cadherin/MMP2/LAMC2 networks	[57]
	VEGF	Angiogenesis	–	[58]
	PDGF-C	Angiogenesis	–	[59]
	PGF	Angiogenesis	–	[60]
Cytokines	CXCL12	Proliferation, migration, invasion	–	[49]
		Induce VM	Activate VE-cadherin/ MMP2/LAMC2 network	[57]
		Immunosuppression	Recruit monocytes and facilitate differentiation into MDSCs in IL-6-STAT3-dependent manner	[61]
	IL-6	Proliferation, migration, invasion	Activate mTOR signaling	[62]
		Immunosuppression	Activate STAT3 signaling	[61, 63, 64]
		Stemness	IL-6/STAT3/notch signaling	[56, 65]
	CCL2, CCL5	Migration	Hh pathway	[66]
	CCL7, CXCL16	Migration, invasion	TGF-β signaling	[66]
	FOXQ1	Initiation	Trans-activate NDRG1	[67]
Extracellular vesicles	Exsomal miR-320a	Suppress proliferation, migration	Inhibit the MAPK pathway	[68]

BMP4, bone morphogenetic Protein-4; ECM, extracellular matrix; COMP, cartilage oligomeric matrix protein; MMP, metalloproteinases; EMT, epithelial–mesenchymal transition; HGF, hepatocyte growth factor; TGF-β, transforming growth factor-β; VM, vasculogenic mimicry; IL-6, interleukin 6; mTOR, mechanistic target of rapamycin kinase; STAT3, activate signal transducer and activator of Janus kinase; LAMC2, Laminin Subunit Gamma 2; VEGF, vascular endothelial growth factor; PDGF-C, platelet derive growth factor-C; PGF, placental growth factor; CCL2/5/7/12, chemokine (C–C motif) ligand 2/5/7/12; CXCL16, chemokine (C-X-C motif) ligand 16; MDSCs, marrow-derived suppressor cells; Hh, hedgehog; FOXQ1, forkhead box Q1; NDRG1, N-myc downstream-regulated gene 1; MAPK, mitogen-activated protein kinase

### CAFs alter ECM in HCC

The ECM is a dynamic system consisted of collagens, elastin, fibrin, and proteoglycans undergoing constant remodeling. Normal ECM benefits resident cells by providing structural and biochemical support [69]. Nevertheless, altered ECM is accompanied with tumor initiation and progression [70]. Current studies indicate that CAFs secrete a large number of ECM proteins for accelerating tumor progression via matrix degradation, deposition, and stiffening [71]. In the TME, activated CAFs secrete ECM proteins to reinforce the deposition of fibrillar collagens, thereby giving rise to the ECM stiffening. Santamato et al*.* reported that through secreting the ECM component laminin-5, activated CAFs stimulated the migration and invasion of HCC cells through the MEK/ERK pathway [72]. In addition to boosting metastasis behaviors, Schrader et al*.* demonstrated that the strengthened tumor matrix stiffness also improved the proliferation of HCC cells through the PKB/Akt pathway [73]. Contractility is considered as another physical function of CAFs involved in ECM restructuring. By exerting a mechanical force either widening the pores in the ECM or aligning collagen fibers, CAFs could “create the path” for cancer cells to migrate and “guide” cancer cells for directional migration [71]. Following the alterations of ECM, CAFs re-establish a favorable stromal environment for cancer cell invasion. Meanwhile, the matrix answers back by activating CAFs to differentiation. This feedback loop is of great significance in maintaining the activated state of CAFs and constructing the tumorigenicity stroma.

### CAFs enhance the stemness of HCC cells

CSC is one specific subset of cancer cells identified in HCC tissues, maintaining a highly adaptable and dynamic state [74]. Manifested in capabilities of proliferation, self-renewal and migration, CSCs facilitate the initiation, growth, and metastasis of HCC. Under specific conditions, CSC characteristics can be triggered by stimuli from TME [75]. Prior studies have noted that the importance of paracrine pathways in the CAFs-induced stem properties of HCC cells. Lau et al*.* elucidated that CAFs-derived HGF expanded liver tumor-initiating cells by regulating c-Met/FRA1/HEY1/ERK cascade [55, 56]. Focusing on CD24 + HCC cells, Li et al*.* emphasized that CAFs induced the stemness via HGF- and IL-6-activated STAT3 pathway in vitro and in vivo [56]. Consistently, Xiong et al*.* also indicated that CAFs-secreted IL-6 promoted the stem properties of HCC cells through STAT3/Notch signaling [65]. According to the latest study, CAFs and activated PTFs were more likely to recruit CSCs and maintain their stemness by producing a series of cytokines, including IL-6, CCL2, CXCL1, CXCL8, SCGF-b, HGF and VEGF [76]. Sun et al*.* demonstrated that CAFs-derived cartilage oligomeric matrix protein (COMP) endowed HCC cells with stem-like properties, accelerating the invasion and metastasis of HCC cells [30]. Furthermore, CAFs could also activate Notch3/LSD1 signaling and autophagy-related mTOR pathway of HCC cells, subsequently driving their self-renewal in CSCs [77, 78]. Interestingly, there might be a potential positive loop between CSCs and CAFs. Huang et al. discovered that CAFs secreted TGF-β to sustain self-renewal of pluripotent stem cells, which conversely maintained CAFs in an active state with higher secretary and proliferate characteristics through the CAF-CSC crosstalk [50]. Collectively, CAFs regulate stem features of HCC cells to foster the development of HCC.

### CAFs promote chemoresistance

Therapy resistance is an undeniable issue for HCC eradication. Though Sorafenib is recommended as preference for systemic therapy, its efficiency is reported only 30% in HCC patients with acquired resistance within 6 months. Mechanisms accounting for drug resistance are complicated, of which tumor-stromal interactions may alleviate the sensitivity of HCC cells to anti-cancer drugs. As elucidated above, CAFs secret multiple types of cytokines and vesicles, thereby inducing the therapy resistance of HCC cells. CAFs-derived HGF enhanced the chemoresistant characteristics of CD73+ tumor cells against sorafenib or cisplatin by activating the MEK-ERK1/2 pathway [79]. Khawar et al*.* found that CAFs—HCC cells—mixed spheroids showed enhanced resistance to sorafenib, while TGF-β inhibitors further improved drug efficacy [80]. Consistently, Liu et al*.* noted that conditioned medium of CAFs conferred HCC organoids resistance to anti-cancer drugs including Sorafenib, Regorafenib and 5-FU in a paracrine signaling-dependent manner [81]. Aronovich et al*.* demonstrated that CAFs protected tumor cells from doxorubicin-induced cell death through secretion of CXCL12, which enhanced chemoresistance by binding to CXCR4 [82]. Interestingly, CAFs-derived CXCL12 might facilitate cisplatin resistance by activating the Wnt/β-catenin pathway [83]. Moreover, Zhang et al*.* found that neuregulin 1 (NRG1) in CAF supernatant promoted resistance in tumor cells by enhancing HER3 expression [84]. In addition, by analyzing on a three-layered microfluidic tumor-on-a-chip platform, CAFs significantly promoted proliferation and delayed doxorubicin pharmacokinetics of tumor cells when compared to coculture with normal fibroblasts [85]. Furthermore, CAFs-extracellular vesicles (EV)-derived Annexin A6 conferred tumor cells resistance against conventional chemotherapeutics by modulating β1 integrin/FAK/YAP signaling [86].

### CAFs facilitate angiogenesis and vasculogenic mimicry in HCC

Tumor angiogenesis refers to the establishment of new blood vessels derived from pre-existing ones. Given that tumor growth and metastasis need nutrients and oxygens, angiogenesis is considered as a hallmark of malignancy, especially for highly-vascularized HCC. Angiogenesis can be regulated by various pro-angiogenic stimulators secreted by cancer cells and stromal cells in the microenvironment. As a major component of tumor stromal, CAFs activated by physiological stimuli like leptin or hypoxia could induce angiogenesis and expedite HCC growth via producing angiogenic factors [87]. Liu et al*.* indicated that CD90 and placental growth factor (PGF) enriched-CAFs were profoundly correlated with tumor angiogenesis markers (CD31, CD34, and CD105) [60]. Moreover, the overexpression of PGF was correlated with angiogenesis markers and poor prognosis [88, 89]. Notably, the crosstalk between HCC and CAFs contributes to angiogenesis as a positive loop. HCC cells fuel CAFs with elevated secretion of the pro-angiogenesis factor vascular endothelial growth factor (VEGF), which enhances the expansion and tumor vessel formation of HCC cells via upregulating EZH2/VASH1 pathway [90]. These evidences suggest the underlying effects of CAFs on the angiogenesis of HCC in vivo and in vitro*.*

Extensive studies have suggested anti-angiogenic drug as an effective anti-tumor strategy, which target vascular endothelial cells to block blood supply to tumor cells. However, angiogenesis may not be the exclusive mechanism that tumors acquire microcirculation. Aggressive tumor cells are capable of forming highly patterned vascular channels and vasculogenic mimicry (VM), in which tumor cells generate their own blood-delivery channels without the participation of endothelial cells [91]. Of interest, Yang et al*.* found that CAFs ratio in VM + HCC tissue was significantly higher than that in VM− tissue. Further investigation disclosed CAFs isolated from fresh HCC tissues remarkably enhanced VM formation in vitro and in vivo through enhancing the expression of VE-cadherin, MMP2 and laminin5γ2 via TGF-βR1 and CXCR4 in HCC cells, suggesting the roles of CAFs in facilitating VM formation and angiogenesis [57].

### CAFs induce immunosuppressive milieu in HCC

Increasing studies highlight the immunosuppressive functions of TME in favoring the HCC progression and potential drug resistance. The initiation of such immune-tolerant microenvironment is mediated by numerous inflammatory factors such as growth factors, cytokines, chemokines generated by multiple cell types co-existed and interacted in TME [92, 93]. Of them, the key constructive cells include TAMs, marrow-derived suppressor cells (MDSCs), tumor-associated neutrophils, CAFs, as well as infiltrating T cells and natural killer (NK) cells [94].

Accumulating studies have emphasized the major role of CAFs in shaping the immunosuppressive TME. By producing immune prohibitive cytokines and immune checkpoint ligands, CAFs recruit immunosuppressive cells like MDSCs and peripheral blood neutrophils, and modulate immune cells differentiation such as monocytes and dendritic cells (DCs) [95]. MDSCs are heterogeneous populations composed of immature myeloid cells with various differentiation states. With the capability to inhibit the proliferation and activity of T and NK cells, MDSCs restrain the immune response of the TME. In a murine model of hepatic cancer, Yang et al*.* found that FAP(+) CAFs-secreted CCL2 recruited circulating MDSCs with high CCL2R expression via STAT3-CCL2 signaling. Conversely, neutralizing anti-CCL2 antibody or knockdown of CCL2 remarkably blocked the migration of MDSCs and abrogated FAP(+) CAF-mediated tumor promotion [96]. In addition, Cheng et al*.* indicated that CAF-derived IL-6 could recruit and regulate the survival, activation, and function of neutrophils through STAT3-programmed cell death ligand 1 (STAT3-PDL1) signaling cascade, afterward contributing to the immune tolerance of HCC cells [64]. According to the study of Li et al*.,* CAFs produced IL-6 could activate STAT3 in DCs, boosting the generation of regulatory DCs, which are characterized by tolerogenic phenotypes with high expression of suppressive cytokines. Moreover, these CAF-educated DCs could confer T cells a suppressive phenotype with the decreased production of IFN-γ in CD8+ T cells and the expansion of CD4+ CD25+ Foxp3+ Tregs expansion [97]. Apart from that, hepatic CAFs could also recruit monocytes by stromal cell-derived factor (SDF)-1a/CXCR4 pathway, and stimulate the monocytes differentiation into MDSCs in IL-6-STAT3-dependent manner, thereby eliminating the anti-tumor immune responses by inhibiting T cell proliferation and functions [61]. Furthermore, HCC derived CAFs inactivated NK cells by secreting indoleamine 2,3‑dioxygenase (IDO) and PGE2, thereby forming an immune-tolerant niche appropriate for HCC progression [97]. Another study reported that CAFs secreted TGF‑β could improve the Tregs proliferation, thus accelerating the HCC growth in the hepatic TME [63]. These evidences demonstrated that CAFs contribute to the immunosuppression functions of HCC via interacting with various types of immune cells in TME.

### CAFs associate with clinical outcome of HCC

The molecular heterogeneity of HCC is manifested in both tumor and tumor stromal cells, triggering distinct clinical outcomes. As CAFs contribute to HCC progression, this key tumor stromal component has the potential to predict the survival of HCC patients. Current studies have found the correlation of α-SMA (+) CAFs with poor clinical outcome in HCC cases. Lau et al*.* reported that α-SMA expression was negatively correlated with disease-free survival (DFS) of HCC patients [55]. Consistent with it, Fang et al*.* also found HCC cases with higher proportion of a-SMA(+) CAFs had shorter DFS [98]. Another study showed that the HCC recipients with α-SMA(+) CAFs had higher risk of recurrence after liver transplantation [99]. Additionally, Yang et al*.* suggested a-SMA(+) CAFs as a biomarker for HCC progression due to the implications in metastasis and tumor staging [100]. At gene level, the encoding gene of a-SMA, ACTA2, was also associated with clinicopathologic features including TNM stage, tumor size, tumor encapsulation, and vascular invasion. Moreover, HCC patients with higher expression of ACTA2 exhibited shorter over survival (OS) and higher recurrent rate [67].

It was recently reported that CAFs-expressed CD90 was associated with clinicopathologic features of HCC. Zhao et al*.* pointed out that high levels of CD90(+) CAFs were correlated with advanced pathological grade, satellite lesion, PVTT, and HCC recurrence. Furthermore, CD90(+) CAFs were indicative of unfavorable outcome of HCC patients after hepatic resection [101]. Other than cell surface markers, CAFs derivations are candidate predictors for HCC prognosis. Liu et al*.* clarified that CAFs-derived PGF, specifically contributing to the neo-angiogenesis, was vastly correlated with unfavorable prognosis of HCC patients [102]. Consistently, Xu et al*.* signified that high expression of PGF in peri-tumor tissues were indicative of poor OS of HCC patients [89]. In addition, the expression of PGF was also positively associated with early recurrence of HCC patients [88]. Significant telomere attrition has been observed in tumor tissues compared to their normal counterparts. Ma et al*.* suggested that shortened telomeres in CAFs resulted in decreased survival time and increased recurrence rate of HCC patients. This finding was further confirmed in an independent cohort from The Cancer Genome Atlas (TCGA) public database [103]. Taken together, the surface molecular markers and derivations of CAFs may provide potential prognosis of HCC outcome.

## Target CAFs in HCC

Given the remarkable tumor-supportive roles, CAFs are evaluated as promising therapeutic targets for cancer intervention. It is generally accepted that cancer cells are liable to develop resistance to various types of therapies due to the genetic instability. Comparatively, CAFs are genetically more stable and less prone to acquire drug resistance [104]. Generally, the strategies of targeting CAFs include targeting specific markers, endowing CAFs with tumor-suppressive phenotype, depleting the CAFs’ precursors, and repressing the secretions or downstream signaling molecules of CAFs. Herein, we summarized current studies regarding CAFs-targeted treatment for HCC (Table 2).

**Table 2:** Strategies of targeting CAFs in HCC

Therapeutic approach	Therapeutic setting	Mechanism	Major effects on HCC	References
Engineer CAFs	miR-335	–	Inhibit proliferation and invasion	[105]
	miR-320	Target PBX3 to suppress MAPK pathway	Inhibit proliferation and metastasis	[106]
	miR-101	Target TGF-β/ SDF1-VE-cadherin/MMP2/LAMC2 networks	Suppress CAFs-promoted VM formation	[57]
Target CAFs’ precursors	Sibrotuzumab	–	Inhibit HSCs activation	[107]
	DFOG	Suppress FOXM1 expression and HGF secretion	Inhibit CSC features and activation of HSCs	[108]
	Metformin	–	Inactivate HSCs and abrogate hepatocarcinogenesis	[109]
	Curcumin	Inactivate ROS/ HIF-1α/CTGF signaling	Suppress HSCs-induced angiogenesis and invasion	[110]
	α-bromomethylene phosphonate- LPA		Block the transformation from PTFs to CAFs	[45]
Target paracrine productions of CAFs	IL-6 neutralizing antibody	Inhibit IL-6 signaling	Deplete stem cell-like properties of HCC cells	[65]
	LY2109761	Inhibit the synthesis and release of CTGF	Reduce HCC growth and dissemination	[47]
	CCL2,5,7 antibodies	Inhibit Hh and TGF-β pathways	Inhibit tumor migration	[66]
	T0901317	Abrogate TGF-β-induced phenotypes through LXRα interactions	Suppress HCC growth	[42]
	RvD1	Suppress COMP secretiom by targeting FPR2/ROS/FOXM1 signaling	Impede CAFs-induced EMT and stemness features of HCC cells	[30]
Target CAFs-mediated signaling and pathways	Dorsomorphin	Inhibit SMAD signaling	Impede the activation of CAFs	[51]
	P38 MAPK inhibitor	Block CAF-M-MDSC crosstalk	Provoke antitumor immunity	[111]
	Rapamycin	Suppress mTOR-signaling pathway	Inhibit HCC cells proliferation, migration and invasion	[62]
	Cryptotanshinone	Inactivate p-STAT3 signaling	Abrogate stem cell-like properties of HCC cells	[65]

COMP, cartilage oligomeric matrix protein; TGF-β, transforming growth factor-β; VM, vasculogenic mimicry; IL-6, interleukin 6; mTOR, mechanistic target of rapamycin kinase; STAT3, activate signal transducer and activator of Janus kinase; CCL2/5/7/12, chemokine (C–C motif) ligand 2/5/7/12; CXCL16, chemokine (C-X-C motif) ligand 16; MDSCs, marrow-derived suppressor cells; Hh, hedgehog; FOXQ1, forkhead box Q1; CTGF, connective tissue growth factor; MAPK, mitogen-activated protein kinase; RvD1, Resolvin D1; MMP, metalloproteinases; EMT, epithelial–mesenchymal transition; VM, vasculogenic mimicry; ROS, reactive oxygen species; LPA, lysophostatidic acid; HCC, hepatocellular carcinoma; CAFs, cancer-associated fibroblasts; HSCs, hepatic stellate cells; PTFs, peritumoral tissue fibroblasts

### Engineer CAFs to acquire tumor-constraining features

Indeed, it is unavoidable to precisely target CAFs without damaging normal cells, which limits the application of marker depletion. Particularly, directly ablating the vital stromal component CAFs may break the homeostasis and inversely exacerbate the disease. Thus, inactivating CAFs into a quiet state or conferring CAFs the tumor suppressor phenotype might be preferable therapeutic approaches. In a CCl4− and alcohol induced liver fibrosis model, most activated myofibroblasts were declined into inactivated HSCs during regression of liver fibrosis by removing etiological agent [112]. In addition, researchers have employed nanoparticles that can be uptake by CAFs to genetically modify CAFs in situ, whereby CAFs may be engineered as a tumor depletion center to persistently release anti-tumor cytokines [113]. In xenograft mice models, the engineered CAFs served as a tumor-directed cytotoxic chemotherapeutic reservoir to trigger the apoptosis of neighboring tumor cells [114]. Interestingly, the apoptosis of adjacent tumor cells reciprocally reverts CAFs to a quiescent state, orchestrating the suppressive microenvironment that is favorable for a second-wave of nano-therapy. Thus, engineered CAFs may function as cytotoxic drugs, providing a new paradigm for tumor therapy.

CAFs communicated with cancer cells partly by extracellular vesicles (EVs) in a paracrine way. Several studies indicated that downregulation of the signals carried by CAF-derived EVs or exosomes contributed to the proliferation and metastasis of hepatoma cells. In contrast, overexpressing certain miRNAs may confer CAFs the tumor-suppressive phenotypes, providing potential options to impede HCC progression. Wang et al*.* reported that downregulation of miR-335 was observed in cancer cells and CAFs, which was beneficial to cancer development. Moreover, miR-335-engineered CAFs acquired the anti-tumor phenotypes against neighboring cancer cells [105]. Furthermore, Zhang et al*.* demonstrated that CAFs-mediated HCC progression was associated with the loss of exosomal miR-320a derived from CAFs. By reprogramming with exogenous miR-320a, CAFs exerted miR-320a-mediated tumor-suppressing effects on HCC cells [106]. However, these strategies for controlling the evolvement of HCC are still limited and remain further pre-clinically investigated.

### Target paracrine productions of CAFs

Aside from the strategies above, another promising strategy is to target CAF-derived cytokines and chemokines. Growing studies have suggested that the potentially targeted value of CAFs-secreted TGF-β in the VM formation [57], tumor growth and invasion [46, 115]. Morén et al*.* indicated that liver X receptors α (LXRα) agonist T0901317 suppressed HCC growth by abrogating TGF-β-induced fibroblastic phenotypes of CAFs and hepatoma cells [42]. Mechanically, T0901317 repressed the transcriptional induction by TGF-β stimulation through LXRα binding to the adjacent DNA motifs of the ACTA2 promoter. Indeed, several pharmacological approaches of impeding TGF-β signaling have been developed to efficiently attenuate aggressiveness of HCC cells. It has been reported that TGF-β signaling in fibroblasts inhibited T cells penetration and impaired the tumor response to anti-PD-L1 agent. Remarkably, co-administration of TGF-β-blocking and anti-PD-L1 antibodies provoked effective anti-tumor immunity and reversed the chemoresistance of anti-PD-L1 agent [116]. In addition, TGF-β inhibitors have been developed and manifested with inhibitory effects on HCC cells. One typical representative TGF-β receptor inhibitor LY2109761 interrupts the cross-talk between HCC cells and CAFs, leading to a significant reduction of HCC growth and dissemination [47]. More applausively, another TGF-β inhibitor LY2157299 is evaluated at phase II clinical trial for HCC patients that fail in sorafenib treatment [117]. Recently, gold nanoparticles (GNPs) were found to alter cell morphology, migration, and molecular markers of CAFs by decreasing the levels of fibroblast activation protein TGF-β1 [118].

Several chemokines containing CCL2/5/7 and CXCL16 were detected highly expressed in CAF-CM. Further investigation showed that these CAFs-derived chemokines facilitated HCC metastasis through activating Hh and TGF-β pathways in HCC cells. The neutralizing antibodies of the chemokines obviously abolished CAFs-induced HCC migration, suggesting that CAFs-generated chemokines are potential therapeutic targets for HCC [66]. Recent data demonstrated that CAFs released soluble CXCL12 into the HCC microenvironment and activated CXCL12/CXCR4/PI3K/AKT signaling of neighboring HCC cells, which subsequently alleviated apoptosis by elevating the BCL-2/BAX ratio. Indeed, downregulating CXCR4 abrogated the anti-apoptotic effects triggered by CAFs, suggesting the underlying role of CXCL12/CXCR4 pathway in CAFs-mediated apoptosis evasion in HCC milieu [49]. Previous studies have noted that the CXCL12-CXCR4 axis could induce FAP(+) CAFs-mediated immunosuppression by excluding CD8+ T cells. In a PDAC model, targeting the CXCL12-CXCR4 pathway by a specific CXCR4 inhibitor AMD3100 induced infiltration of CD8+ T cells into the tumors. Besides, CXCL12-induced T cell exclusion greatly hindered the anti-tumor effects of anti-PD-L1 monoclonal antibodies, thus AMD3100 synergized with α-PD-L1 could greatly enhance the therapeutic effects on tumor cells [119]. However, the efficiency of targeting CXCL12-CXCR4 axis remain further evaluation in HCC.

Sun et al*.* indicated that Resolvin D1(RvD1), an endogenous anti-inflammatory lipid mediator, impeded CAFs-induced EMT and stemness features of HCC cells by suppressing the secretion of COMP [30]. RvD1 also impaired CAFs-derived COMP in a paracrine manner by targeting FPR2/ROS/FOXM1 signaling, ultimately blocking the FOXM1 recruitment to the COMP promoter. As one of the most abundant cytokines secreted by CAFs in HCC, IL-6 modulates immune response by regulating the generation of DCs, recruitment and function of neutrophils, and the induction of monocytes to differentiate into MDSCs. Recent studies showed that that blocking IL-6 enhanced antitumor immunity in HCC. IL-6 inhibitors could also reverse the anti-PD-L1 resistance of HCC. Moreover, synergized IL-6 and PD-L1 blockade effectively inhibited HCC growth in vivo. In a mice model of HCC, combinational treatment of IL-6 blockade and anti-PD-L1 presented effectiveness with smaller tumor size and longer survival time [120]. In addition, Xiong et al*.* noted that IL-6 neutralizing antibody depleted stem cell-like properties of HCC cells through inactivating STAT3/Notch signaling [65].

As is generally acknowledged that hepatic fibrosis greatly promotes occurrence of HCC, alleviating fibrosis may actually attenuate hepatocarcinogenesis. Accumulating clinical evidence have endorsed the efficiency of low-dose metronomic (LDM) chemotherapy regimen than traditional chemotherapy. Traditional maximum-tolerated dose chemotherapy could unexpectedly induce an ELR+ chemokine–producing phenotype in CAFs, whereby fostering chemoresistance and tumor progression. On the contrary, continuous LDM therapy largely downregulates therapeutics-induced CAFs paracrine signaling and prevents CAFs activation, resulting in an enhanced treatment response [121]. Currently, the effectiveness of either LDM chemotherapy alone or in combination with targeted therapeutics has been validated in several clinical trials [122]. Thus, it is a worthwhile potential approach to overcome CAFs-induced aggressive behaviors in HCC.

### Deplete CAFs by targeting surface markers

α-SMA is a typical marker of the myofibroblast subset of CAFs. However, the effects of targeting α-SMA remains controversial on tumor progression. In a mouse model of breast cancer, targeting α-SMA(+) CAFs obviously impeded tumor metastasis [123]. In contrast, blocking α-SMA enhanced the infiltration of CD3+ Foxp3+ Tregs in tumors, thereby facilitating immunosuppressive TME for tumor progression [124]. Currently, the major strategy for depleting CAFs is mainly targeting another CAF surface marker FAP. Lang et al*.* constructed a CAF-targeting siRNA delivery system by loading the FAP-α antibody onto the cell-penetrating peptide-based nanoparticles, which specifically downregulated CXCL12 expression in CAFs [125]. Polyphyllin I could considerably inhibit FAP, SDF-1, and HGF in CAFs, and further suppress the growth of gastric cancer in vivo [126]. Genetic depletion of FAP reduced the infiltration of FAP(+) CAFs with increased infiltration of CD8+ T cells [127]. In addition, targeting FAP by DNA vaccines efficiently promoted CD8+ T cell-mediated repression of CAFs, thereafter elevating intra-tumor chemotherapeutic drug uptake in multi-drug-resistant tumors [104]. To date, pre-clinical studies suggested targeting FAPα might be efficient approaches, including DNA vaccine, enzymatic inhibitor, neutralizing antibody, and chimeric antigen receptor T-cells [128]. Different from these strategies, Zhen et al*.* conducted a nanoparticle-based photoimmunotherapy that can selectively kill CAFs without causing systemic toxicity [129]. By using FAP-specific single chain variable fragment, this nano-approach efficiently eliminated CAFs and caused tumor suppression in tumor-bearing immunocompetent mice.

### Target precursors of CAFs

Except for targeting CAF cell surface markers, alternative approach of targeting CAFs’ precursors may efficiently reduce the generation of CAFs. Remarkably, as the main source of CAFs in HCC, the activated HSCs can be targeted by sibrotuzumab without toxic to normal hepatic cells [107]. Additionally, Chen et al*.* demonstrated that a genistein derivative DFOG intervened the crosstalk between HSCs and hepatic CSCs by downregulating FOXM1 expression and HGF secretion of HSCs. Metformin, a well-known anti-diabetes drug, was previously reported to prevent liver tumorigenesis by attenuating HSCs activation in CCl4 challenged transgenic mouse model [109]. Another widely used agent curcumin could significantly suppress the HSCs-induced aggressive behaviors of HCC cells by inhibiting reactive oxygen species (ROS)/HIF-1α/CTGF signaling [110]. Currently, activated HSCs can also be inhibited by efficient anti-fibrotic drugs, including PRI-724, follistatin, Gliotoxin, salvianolic acid, sulfasalazine, Curcumin, tanshinone I, and conophylline [130–133]. It is worth noting that these drugs against liver fibrosis may also benefit for HCC treatment by blunting of CAFs’ activation [94]. Apart from HSCs, PTFs might also be the candidate target. A pan-LPA inhibitor (α-bromomethylene phosphonate-LPA) blocked the transformation from PTFs into CAFs, subsequently suppressing HCC growth and progression in vitro and in vivo [45]*.*

### Block activation signaling and downstream effectors of CAFs

Monocytic MDSCs (M-MDSCs) accumulated in fibrotic livers, which is associated with decreased tumor-infiltrating lymphocytes (TILs) and increased tumorigenicity in mouse models. In human HCC, M-MDSCs enriched in the para-cancerous fibrotic liver tissues are remarkably correlated with aggressive tumor phenotypes and shorter survival. Prior study indicated that M-MDSCs in HCC could be differentiate from monocytes activated by CAFs through monocyte-intrinsic p38 MAPK signaling. Interestingly, targeting the CAF-M-MDSC crosstalk using p38 MAPK inhibitor significantly enhanced the efficacy of anti-PD-L1 therapy and led to tumor eradication, ultimately prolonging survival in the fibrotic-HCC mouse model [111].

Almost half of the HCC cases show upregulated activity of mTOR pathway, which plays essential roles in tumor growth, proliferation and apoptosis. Since CAFs-derived factors trigger hepatocarcinogenesis partially through mTOR cascade signaling, inactivating mTOR signaling may be a potential treatment for HCC. Indeed, the mTOR signaling pathway inhibitor rapamycin exhibited antitumoral activity by interfering with progranulin or IL-6-mediated proliferation and invasion of HCC cells [62]. In lung cancer and melanoma, the IGF2 neutralizing antibody and the autophagy inhibitor 3-MA dramatically reduced the CAF-promoted tumor relapse in mice after radiotherapy by regulating m-TOR-mediated autophagic activities. Additionally, mTOR pathway contributes to the synthesis of various secretions of α-SMA(+) CAFs, thereby eliminating the CAF-mediated drug resistance in cancer cells. Therefore, targeting mTOR signaling attenuates the production of multifarious secretions, which provide an alternative option for cancer treatment [134]. As described above, BMP4 activated NFs into CAFs via SMAD signaling pathway. Further dorsomorphin (SMAD 1/5/8 inhibitor) treatment suppressed the elevation of ACTA2 and IL-6 induced by exogenous BMP4 [51]. Aside from that, p-STAT3 (Tyr705) inhibitor cryptotanshinone could also deplete the CAFs-induced CSC effects on HCC cells by abrogating STAT3 signaling [65]. These evidences suggest that the key genes and signaling pathways involved in the activation of CAFs are promising molecular targets for HCC treatment.

## Conclusion

Accumulating evidences suggest the pivotal roles of CAFs in favoring aggressive behaviors in HCC. In this review, we explicitly summarized the heterogeneity of CAFs in HCC, regarding cell origin, location, and phenotypes. Potential mechanisms by which CAFs fuel HCC progression include ECM remodeling, neovascularization, immunosuppression, EMT and stemness of HCC cells. Given the crucial roles in HCC progression, CAF may be an attractive target for the treatment of HCC. Growing studies have highlighted the benefits of “anti-CAFs” therapy for tumor patients. However, for the current status, it is formidable to target CAFs precisely without damaging normal tissue due to the elusive sources and less specific markers. Additionally, considering the existence of cancer-restraining CAFs in other cancer types, the exact roles of CAFs in HCC should be further determined [135, 136]. In conclusion, it is attached great significance to further investigating the roles and mechanisms involved in the CAFs in HCC progression, which will provide candidate targets for HCC treatment.

## Data Availability

Not applicable.

## Abbreviations

HCC: Hepatocellular carcinoma
HCV: Hepatitis C virus
NAFLD: Non-alcoholic fatty liver disease
HBV: Chronic hepatitis B virus
NASH: Non-alcoholic steatohepatitis
ALD: Alcohol-related liver disease
IR: Insulin resistance
HSCs: Hepatic stellate cells
ECM: Extracellular matrix
TKI: Tyrosine kinase inhibitor
OS: Over survival
PME: Premalignant microenvironment
TME: Tumor microenvironment
CAFs: Cancer-associated fibroblasts
TAMs: Tumor-associated macrophages
VM: Vasculogenic mimicry
ER: Endoplasmic reticulum
ED-A: Ectodysplasin-A
EGF: Epidermal growth factor
FGF: Fibroblast growth factor
HGF: Hypoxic growth factor
PDGF: Platelet derive growth factor
CXCL12: Chemokines like chemokine ligand 12
TGF-β: Transforming growth factor-β
EndMT: Endothelial-to-mesenchymal transition
EMT: Epithelial–mesenchymal transition
α-SMA: α-Smooth muscle actin
FAP: Fibroblast activation protein
FSP1: Fibroblast specific protein 1
PDGFR: Platelet-derived growth factor receptor
PDAC: Pancreatic ductal adenocarcinoma
MSCs: Mesenchymal stromal cells
HSECs: Hepatic sinusoidal endothelial cells
PTFs: Peritumoral tissue fibroblasts
HIF-1α: Hypoxia inducible factor 1α
LPA: Lysophostatidic acid
PFs: Portal fibroblasts
LXR: Liver X receptors
CTGF: Connective tissue growth factor
CSCs: Cancer stem cells
TAFs: Tumor tissue-derived fibroblasts
COMP: Oligomeric matrix protein
VEGF: Vascular endothelial growth factor
PGF: Placental growth factor
MDSCs: Marrow-derived suppressor cells
Tregs: Regulatory T cells
MDSCs: Myeloid-derived suppressor cells
STAT3-PDL: Activate signal transducer and activator of Janus kinase-programmed cell death ligand 1
LDM: Low-dose metronomic
CM: Conditioned medium
EVs: Extracellular vesicles
DFS: Disease-free survival
